# Inpatient Rehabilitation during Intensive Refeeding in Severe Anorexia Nervosa

**DOI:** 10.3390/nu14142951

**Published:** 2022-07-19

**Authors:** Marc Dauty, Pierre Menu, Baptiste Jolly, Sylvain Lambert, Bruno Rocher, Maëlle Le Bras, Adam Jirka, Pascale Guillot, Stéphane Pretagut, Alban Fouasson-Chailloux

**Affiliations:** 1 Service de Médecine Physique et Réadaptation Locomotrice et Respiratoire, CHU Nantes, Nantes Université, 44093 Nantes, France; marc.dauty@chu-nantes.fr (M.D.); pierre.menu@chu-nantes.fr (P.M.); baptiste.jolly@chu-nantes.fr (B.J.); 2 Service de Médecine du Sport, CHU Nantes, Nantes Université, 44093 Nantes, France; stephane.pretagut@chu-nantes.fr; 3 IRMS, Institut Régional de Médecine du Sport, 44093 Nantes, France; 4 Inserm UMR 1229, Regenerative Medicine and Skeleton, RMeS, Nantes Université, ONIRIS, 44042 Nantes, France; 5 Psychiatrie et Santé Mentale, UIC 18, CHU Nantes, Nantes Université, 44000 Nantes, France; sylvain.lambert@chu-nantes.fr (S.L.); bruno.rocher@chu-nantes.fr (B.R.); 6 Service d’Endocrinologie, Diabétologie et Nutrition, Institut du Thorax, CHU Nantes, Nantes Université, 44000 Nantes, France; maelle.lebras@chu-nantes.fr; 7 Equipe Transversale D’assistance Nutritionnelle, CHU Nantes, Nantes Université, 44000 Nantes, France; adam.jirka@chu-nantes.fr; 8 Service de Rhumatologie, CHU Nantes, Nantes Université, 44000 Nantes, France; pascale.guillot@chu-nantes.fr

**Keywords:** anorexia nervosa, body mass index, physical activities, muscle strength, hyperactivity

## Abstract

Severe forms of anorexia nervosa are responsible for weight loss and life-threatening consequences. Refeeding represents a real psychiatric and somatic challenge. Physical activities are usually not recommended during intensive refeeding in order to avoid energy expenditure. This study assessed the interest in an early return to controlled physical activities, during a hospitalization in a Physical Medicine and Rehabilitation (PMR) department, including continuous nasogastric refeeding and psychiatric care. A total of 37 subjects aged 32 ± 11 years old performed inpatient physical activities during nasogastric refeeding initiated after intensive care. The physical activity program was adapted according to the hyperactivity of the patients. Evaluation parameters were weight, body mass index (BMI), body composition (fat, lean, and bone masses), and function (strength, balance, walking, ventilation). Patient satisfaction, re-hospitalizations, and physical activities continuation were assessed at 12 months of follow-up. Weight, BMI, and body fat increased significantly (+2.7 ± 1.7 kg; +1.0 ± 0.6 kg/m^2^; +1.7 ± 2.5 kg, respectively). Muscle strength increased even if the lean mass did not. Walking distance, balance, and respiratory function were significantly improved. Weight and fat mass gains did not differ according to the presence or absence of hyperactivity. At 12 months, 46% of the patients continued to be physically active, but 21% of the patients had been re-hospitalized. The early return to controlled physical activities in PMR hospitalization does not compromise the efficiency of intensive refeeding in severe anorexia nervosa patients.

## 1. Introduction

Anorexia nervosa is a psychiatric illness that mainly affects young women aged 15 to 19, with an incidence between 4.2 and 8.3/100,000/year [1,2]. The disease evolution is variable, from recovery to chronic forms with the possibility of death due to ionic disorders, infection, or suicide (0.5% a year) [3,4,5]. Patients with serious forms of malnutrition are hospitalized for continuous nasogastric refeeding because of severe somatic conditions requiring a multidisciplinary management [6,7,8,9,10]. Weight loss is often associated with a loss of strength and an increase in daily-life dependency [11,12]. The management has several objectives, with the increase in weight and Body Mass Index (BMI) as benchmarks, reflecting the clinical improvement of the patient [8,11]. For severe forms of anorexia nervosa, a period of continuous nasogastric refeeding carried out in intensive care is usually necessary in order to correct ionic and energy imbalances [13]. Then, returning home can be considered, but there are risks of recurrence [8]. Alternatives to hospitalization most often require oral nutrition to be restarted [8]. Hospitalization in the Physical Medicine and Rehabilitation (PMR) department has the ability to offer physiotherapy care and adapted physical activities to help the patient recover strength and autonomy (e.g., walking, sit-to-stand without assistance) while continuing intensive refeeding [14]. The practice of physical activities was first proposed in 1970 by Blinder et al. [15]. A weight gain of 3.9 pounds per week (+1.75 kg) was obtained in patients during a stay of 6 weeks, which included the practice of resistance exercises. Since then, different meta-analyses have shown that muscle strength and function were improved without negative effects on anthropometric measurements (body weight and BMI) in the case of calorie intake adjustment [16,17,18]. The practice of physical activities could also improve body perception, eating disorder symptoms, mood, and quality of life [14,17,19,20]. However, controlling physical activities is essential because of the malnourished state of the subjects, who may also present hyperactivity [21]. This hyperactivity can be intentional so as to lose weight and control body weight, or unintentional but due to obsession compulsions, which poses the problem of the dependence on physical activity, accompanied by an anxious state or even depression if these activities are no longer practiced [22,23]. Conversely, physical activity can be used to reduce anxiety [24]. Hyperactivity is present in 30 to 75% of the anorexic subjects and is responsible for difficulties in refeeding, risk of drop-out during treatment and recurrences [25]. Therefore exercise has been considered a behavior problem and is usually forbidden during refeeding. The goal of PMR hospitalization is, therefore, to continue nasogastric refeeding and psychiatric care while performing controlled and adapted physical activities.

The main objective of this study was to assess if this original treatment, performed during intensive nasogastric refeeding, improved muscle strength and patients’ autonomy. The secondary objectives were to compare the results depending on the presence or absence of hyperactivity and to know if physical activities were safe, and then, if safe, continue them over a 12-month period.

## 2. Materials and Methods

### 2.1. Population

We included patients from 2015 to 2020 addressed for hospitalization in the PMR department. Subjects had to present anorexia nervosa. They were hospitalized following a stay in intensive care for refeeding. The diagnosis of anorexia nervosa was made by a psychiatrist specializing in eating disorders according to the DSM-V classification of the American Psychiatric Association [26].

Before entering PMR hospitalization, any ionic disorder and hepatic cytolysis had been corrected during the hospital stay in intensive care. In addition, the patients had to give their consent to perform physical exercises during refeeding after consulting the psychiatrist. A three-partite contract (Patient, PMR Physician, and Psychiatrist) for a renewable period of 4 weeks was signed at the entry into the PMR inpatient care. Patients who presented a weight loss unrelated to anorexia nervosa or a psychiatric condition contraindicating PMR care were not included. Patients with vestibular and cerebellar disorders, polyneuropathy, or musculoskeletal traumatism, as well as asthmatic respiratory disease or smokers, were excluded.

All the patients gave written consent to participate in the study. The local ethics committee (Comité Nantais d’Ethique en Médecine du Sport) approved the protocol under the number of registration CNEMS-2022-001. The study was also declared to the Research Department of the University Hospital. The study was in accordance with the declaration of Helsinki [20].

### 2.2. Contracted Support for PMR Management

The objectives of the PMR care were (1) to continue the weight gain of more than 500 g a week with weighing carried out twice a week, (2) to stop the nasogastric tube feeding gradually (intake at the entry of PMR >1500 Kcal/day) according to a reduction of 250 Kcal per week if the weekly objective of weight gain was reached. On discharge, oral feeding should be exclusive, and (3) to perform controlled physical activities to improve strength, balance, respiratory function, and walking autonomy. Cardiac contraindications were previously assessed: hypotension <80/50 mmHg, bradycardia <50 beats/min, arrhythmia, QRS abnormalities [27].

### 2.3. Program of Physical Activities

The exercise program was developed based on the results of Ng et al. and Moola et al. in 2013 [16,17]. This program was monitored 3 times a week by a PMR physician and controlled by a physiotherapist and a sports instructor. The first week was devoted to the somatic evaluation, which allowed the initiation of physical activities for 1 h a day and to determine whether the patient presented signs of hyperactivity or not. Due to multiple definitions of hyperactivity in anorexia nervosa [25], the diagnosis of hyperactivity was made if the subject attempted to perform exercises in addition to the controlled exercise requested.

During the following weeks, 1-h sessions were performed twice a day, 5 days out of 7, with a 10-min warm-up and a 10-min cool-down. The morning was dedicated to muscle stretching exercises of the upper and lower limbs, postural maintenance of the trunk (started by 15 s of maintenance targeting stiffness and gradually increased to 30 s × 2 sets), balance (monopodal support: eyes open then closed, for 10 s), and relaxation including breath control with thoracic expansion work [14]. Twice a week in the afternoon, physical exercises for progressive muscle strengthening were performed according to the somatic evaluation. Isometric exercises were performed for the muscles of the trunk, and dynamic exercises with elastic bands were performed for the muscles of the limbs (concentric muscle contractions for the upper limbs and eccentric contractions for the lower limbs, from 6 to 10 repetitions, 3 to 5 sets with recovery of 1 min between sets [11]. Functional exercises included getting up from the floor, chair lifting, walking, and stair practice according to the objective of patient autonomy.

Adapted physical activities were performed 3 times a week, on Mondays, Wednesdays, and Fridays, including 1 h of badminton, initially practiced in a sitting position, then standing, and table tennis, billiards, or archery. Volleyball in the pool and swimming were also proposed, as well as the practice of walking and cycling at the end of the stay according to the weight gain. The exercises were intermittent from 1 to 3 min with a recovery period of at least 3 times the exercise duration (the heart rate should have dropped to less than 120 beats per minute) without exceeding a total practice time of 30 min. A cool-down was then performed with stretching. If the weight goal was not reached or if there was uncontrolled hyperactivity, afternoon physical activities were replaced by a mandatory 1-h rest period followed by a 1-h session of relaxation in hot water balneotherapy (32–34 °C).

The last week was devoted again to the somatic assessment. The length of hospitalization in PMR and the duration of nasogastric refeeding were reported in days, as well as the tolerance to physical activities.

### 2.4. Psychiatric, Dietetic, and Biological Follow-Up

Throughout the PMR department stay, the patients were followed once a week by a psychiatrist in order to adapt the psychotropic treatment if needed, to provide psychological support, and to help control their weight and possible hyperactivity.

A dietary consultation was performed once a week after medical advice in order to continue the treatment with vitamin and magnesium therapy and gradually introduce oral food, taking into account the reduction of calories provided by the nasogastric tube. The dietician also provided information and advice about nutrition, such as the role of the necessary nutrients for physiological functioning and the need for sufficient nutritional intake in order to achieve the objectives of the hospitalization. The meal times were supervised by a nurse to prevent the possibility of self-induced vomiting.

Various biological parameters were monitored in order to detect a phosphor-calcium disorder (calcium, phosphorus, protein, Vitamin D, and Parathormone) and an inappropriate refeeding syndrome (Hypophosphatemia, liver cytolysis) [9,13,28].

### 2.5. Somatic Evaluation in the PMR Department

Weight gain was measured to the nearest 0.1 kg twice a week on Monday and Thursday mornings, with an empty stomach and naked (Bathroom scale, SECA 876^®^, Semur en Auxois, France). The Body Mass Index (BMI) was calculated using the weight-to-height-squared formula. The definition of undernutrition according to the World Health Organization was considered grade V for a BMI < 10 kg/m^2^, grade IV for a BMI between 10 and 12.9 kg/m^2^, grade III for a BMI between 13 and 14.9 kg/m^2^, grade II for a BMI between 15 and 16.9 kg/m^2^, and grade I for a BMI between 17 and 18.4 kg/m^2^. Only the weight and the BMI at the entry into intensive refeeding care and at the entry and the discharge PMR department were presented in order to assess the evolution of these two parameters.

Knee muscle strength (Extensors and Flexors was measured in a concentric mode using an isokinetic dynamometer (Humac Norm^®^ Medimex, Sainte-Foy-Les-Lyon, France) at an angular speed of 60°/s (3 repetitions) after a warm-up of 5 repetitions performed at 180°/s. The peak force was expressed in Nm/kg. The average of both sides, right and left, was considered. The reproducibility of the measurements is considered good in healthy subjects (ICC: 0.85–0.99) [29]. The muscle strengths of the biceps and triceps brachii were measured elbow flexed at 90° using an isometric dynamometer (MicroFET2^®^, Biometrics, Orsay, France) to the nearest kg. The results were related to the subjects’ weight at the time of the assessment, and the average of both sides was taken into account. The reproducibility of the measurements is considered good in an elderly population (ICC: 0.74–0.85) [30]. The grip strength was measured using a dynamic dynamometer (Jamar^®^, Madrid, Spain) to the nearest kg. The results were related to the subjects’ weight at the time of assessment, and the average of both sides was considered. The reproducibility of the measurements is considered good in healthy or anorexic subjects [31,32]. Endurance of the trunk muscles was measured with a Shirado–Ito test for the abdominal muscles and a Biering–Sorensen test for the spine muscles [33,34]. The Shirado–Ito test was performed in the supine position, maintaining a hip flexion angle of 60° as long as possible (seconds), knees bent at 90°, feet resting on the ground, and upper limbs crossed on the chest. This test was stopped when the patient could no longer maintain this posture or after 180 s. The reproducibility of this test is considered good (ICC: 0.90) [35]. The Biering–Sorensen endurance test was performed in the prone position, trunk in a vacuum, upper limbs crossed over the chest, pelvis and lower limbs held on an examination table. The subjects had to maintain a horizontal posture for as long as possible (seconds). This test was stopped when the patients could no longer maintain this posture or after 180 s. The reproducibility of this test is considered good in healthy subjects (ICC: 0.62–0.93) [36].

The standing balance, eyes open and then eyes closed, was measured using a stabilometric platform (SATEL^®^, Blagnac, France) focusing on the excursion (sway length in mm) of the center of mass during 25 s, which corresponded to the stabilization of the total body mass exploring the postural responses [37]. These measurements have good reproducibility in healthy subjects (ICC: 0.45–0.86) [38].

The distance (m) achieved during a 6-min walking test was measured during the first and last week of hospitalization in the PMR department under the supervision of a sports instructor [39].

Functional respiratory assessments were performed using a spirometer (Pony FX^®^, Cosmed, Rome, Italy). The maximum expiratory volume per second (FEV1 in L/s) and the Forced Vital Capacity (FVC in L) were measured according to the recommendations of the American and European Respiratory Society (ATS/ERS) [40]. The FEV1/FVC ratio was calculated to assess restrictive or obstructive pulmonary abnormalities.

Hip bone mineral density (Z-score and T-score) was performed with a densitometer (Lunar Prodigy Advance^®^, GR Healthcare, Chicago, IL, USA). Results were reported if this assessment had been performed in the year prior to the hospitalization in PMR in order to assess the bone status [41]. To quantify the bodily effects of the treatment in PMR, the fat and lean masses and the bone mineral content in grams were also measured at the level of the whole body, the limbs, and the trunk before and after PMR hospitalization.

The patients were contacted by phone at 12 months in order to know their opinion on the inpatient PMR management and to know if the physical activities were continued. In addition, the number of patients readmitted for a disorder related to anorexia nervosa was collected.

### 2.6. Statistical Analysis

Statistical analyses were performed using SPSS 23.0^®^ software (Armonk, NY, USA). Quantitative parameters were presented as mean and standard deviation. The normal distribution of the parameters was verified by the Kolgomorov–Smirnoff test. The exercise program was evaluated by a pre/post comparison using a paired *t*-test. The parameters of the subjects who did not complete the first 4 weeks of the contract were included as long as they had been hospitalized for more than 15 days. The comparison between the two subpopulations, hyperactive and non-hyperactive, was performed using a Student’s *t*-test or a Mann–Withney test according to the normal distribution of the variables. A repeated measure analysis of variance was carried out in order to compare the evolution of the weight and the BMI of the hyperactive and the non-hyperactive subjects taking into account these variables at the entry into intensive care, at the entry, and at the exit of the PMR department. The assumption of sphericity was assessed and corrected using the epsilon of Greenhouse–Geisser. Effect sizes were assessed by partial eta squared η2, which were defined as trivial, small, moderate, and large for values η^2^ ≤ 0.1, ≤0.3, ≤0.5, and >0.5, respectively. To assess if the improvement of the parameters during hospitalization in PMR was associated with weight gain, a two-tailed Pearson coefficient correlation analysis was performed. The significance level was determined at *p* < 0.05.

## 3. Results

### 3.1. Evolution of the Population during the Stay in PRM

We included 37 patients aged 32 ± 11 (34 women and 3 men) hospitalized following a stay in intensive care for refeeding of 21.0 ± 12.0 days. They had been presenting anorexia nervosa since the age of 18.4 ± 5.0 years (duration of anorexia of 13 ± 12 years, from 1 to 36 years). At the entry into intensive care, patients had a weight of 33.9 ± 5.3 kg for a height of 160 ± 8.5 cm [146–190], i.e., a BMI of 12.6 ± 1.5 kg/m^2^. Osteopenia or osteoporosis was measured before hospitalization (Table 1), and calcium-vitamin D deficiencies were gradually corrected (Table 2). In total, 13 patients presented with pure restrictive anorexia, 9 had anorexia with purgatory behavior, 11 had anorexia with a history of bulimic episodes, and 4 had anorexia with hyperactivity. Hyperactivity was seen in 17 patients (46%). All the female patients were in amenorrhea and were not taking hormone replacement therapy. Concerning the other parameters, there was no difference according to the sex of the patients.

At the entry into the hospitalization of PMR, the weight was 37.1 ± 5.7 kg for a BMI of 13.8 ± 1.4 kg/m^2^. The duration of refeeding by nasogastric tube was 36 ± 16 days during the hospitalization in PMR. The length of stay in the hospital was, on average, 42 ± 18 days. Four patients were discharged between the third and the fourth week for non-compliance with the contract, and one was retransferred to acute care for refeeding syndrome and then returned to PMR hospitalization.

The mean weight gain was 2.7 ± 1.7 kg [95% CI: 2.2–3.2] (*p* < 0.0001). The mean BMI gain was 1.0 ± 0.6 kg/m^2^ [95% CI: 0.8–1.2] (*p* < 0.001). Total fat mass increased by 1.7 ± 2.5 kg [95% CI: 0.9–2.6] and body fat percentage increased from 6.9 ± 4.3 to 11.0 ± 8.6% (*p* < 0.02). Fat mass increased significantly in the trunk and upper and lower limbs (40%, 29%, and 47%, respectively) while lean mass did not significantly increase and bone parameters remained unchanged (Table 3).

Strength increased significantly in the upper and lower limbs, as well as the endurance in the trunk (Table 4). The walking distance during the 6-min test increased significantly by 90 m (*p* < 0.01), as did the parameters of the balance tests, especially with eyes closed. Respiratory function was significantly improved with a gain in FVC of 200 mL (*p* < 0.01), an improvement of the theoretical values which increased from 82.6 ± 16.4% to 89 ± 16% (*p* = 0.004). The FEV1 also increased (*p* = 0.02), explaining an unchanged FEV1/FVC at 87%, considered non-pathological.

Tolerance to supervised physical activities was good. No stress fracture secondary to supervised physical activity was found in three patients who complained of transient musculoskeletal pain (less than 3 days) related to unusual muscle strain.

### 3.2. Comparison between Hyperactive and Non-Hyperactive Patients

**Figure 1:**
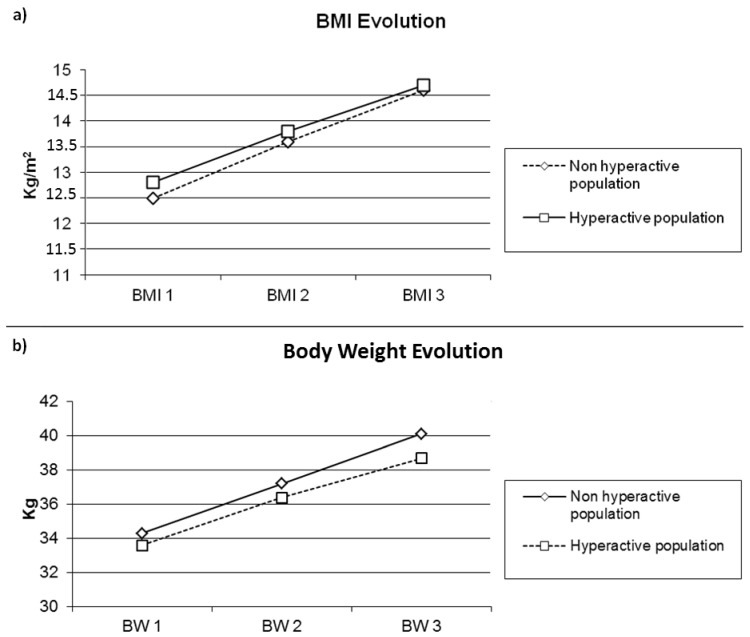
(**a**) Body Mass Index evolution and (**b**) Body Weight evolution according to hyperactivity or not in patients with anorexia nervosa. Abbreviations: BW: Body weight; BMI: Body Mass Index; Evolution 1: Enter in hospital; evolution 2: Enter in PMR; Evolution 3: discharge of MPR.

The population of the 17 hyperactive patients was not different from the non-hyperactive one (*n* = 20) concerning the duration of hospitalization (43.3 ± 18.3 vs. 40.7 ± 18.2 days; *p* = 0.66) and the duration of refeeding by nasal-gastric tube (35.9 ± 17.3 vs. 36.0 ± 15.3 days; *p* = 0.98) (Table 5). The changes in weight and BMI over time were comparable (Weight: F(2.68) = 0.26, *p* = 0.67, η^2^ = 0.008); (BMI: F(2.68) = 0.10, *p* = 0.83, η^2^ = 0.003) (Figure 1a,b). The gains in isometric and isokinetic strength of the limbs and the trunk were comparable, as were gains in balance and gait parameters on the 6-min walking test. The gains of FVC and FEV1 were also no different.

### 3.3. Correlations between Clinical Parameters and Weight Gain

Improvement in the 6-min walking test was associated with weight gain (r = 0.432; *p* < 0.01). There was a tendency for an association between weight gain and isokinetic strength gain in the extensors and flexors of the knees (r = 0.392; *p* = 0.06 and r = 0.374; *p* = 0.07, respectively). Improvement in grip strength, endurance of the trunk, isometric strength of the lower and upper limbs, and respiratory function were not associated with weight gain.

### 3.4. Follow-Up after PMR Hospitalization

The subjective evaluation performed at 12 months of the hospitalization in the PMR department showed that 21 patients were very satisfied (*n* = 6) or satisfied (*n* = 15) because of the medical and paramedical support. Patients reported that this support helped them recover weight, while they perceived progress with a feeling of having more strength. Some of them would have liked the care to last longer, while others felt the care was very strict. Seven subjects were dissatisfied because of the constraints related to the nasogastric tube and the “infantilization” related to the strict framework imposed for weighing.

A total of 17 patients (46%) continued physical activities such as cycling, jogging, fitness, swimming, walking, dancing, and gentle gymnastics. However, among them, 1 patient remained hyperactive (cycling 200 km per week), and another restrictive anorexic patient became hyperactive (running more than 5 times a week). Eleven patients no longer performed physical activities after the PMR treatment. Nine patients did not give their opinion (six non-responders and three dead, including a man and a woman by suicide and a woman by pneumonia).

A total of 8 subjects (21%) were re-hospitalized in the psychiatric service for recurrence, including 6 hyperactive patients. Two patients, a pure restrictive anorexic and an overactive anorexic with purging, were re-hospitalized for consolidation of weight gains.

## 4. Discussion

Adapted and controlled physical activities during hospitalization in a PMR department were performed safely in severe anorexic subjects (BMI < 14 kg/m^2^) with intensive refeeding by nasogastric tube to allow the control of caloric intake. This management required multidisciplinary skills with weekly monitoring of psychiatric care, nutrition, and PMR in order to improve muscle strength, respiratory parameters, balance, and walking while restoring body weight and resuming exclusive oral feeding. Interestingly, we found no differences between the hyperactive and non-hyperactive patients. To our knowledge, this work was the first to propose physical activities during nasogastric refeeding with multiple pre- and post-physical activity assessments while differentiating hyperactive patients from non-hyperactive patients.

Several studies have already shown that it is possible to perform physical activities for anorexic subjects while gaining weight or improving BMI [42,43,44,45,46]. However, no difference has been found compared to control anorexic subjects not performing physical activity [47]. Body fat increase had already been shown in patients with anorexia from Australia aged from 16 to 19 years old (BMI from 14.8 to 15.4 kg/m^2^) who gained between 2.5 and 8% of body fat while they performed various anaerobic and aerobic physical activities [48,49]. According to their measurements by DXA, body fat increased in the lower limbs (47%), the trunk (40%), and the upper limbs (29%) in different proportions from those we have shown, certainly due to the predominance of women in our population. Another hypothesis is that the practice of physical activities played a role in the distribution of fatty masses according to the particular demand of certain regions of the body.

One of the objectives of practicing physical activities during refeeding is to gain lean mass by performing muscle strengthening using the anaerobic energetic system [50]. Lean mass gain had previously been reported after calculation and not by direct measurement [49,50]. However, our measures of lean mass by DXA were unmodified at the end of the hospitalization. We certainly did not show an increase in lean mass because a gain in muscle mass undoubtedly requires more time, 20 to 24 weeks of resistance training at a frequency of twice a week in order to allow the synthesis of muscle proteins [51]. Russel et al. used a physical activity program for 10 to 15 weeks [49]. In fact, in healthy Caucasian subjects, the gain in muscle mass is variable depending on the individual (mean gain of 4.8% from −10 to +30%) with the notion of high and low responders (30% of subjects), without the possibility of predicting lean mass gain according to the BMI, the age, and the sex [51]. The relationship between muscle mass and strength is also weak (r = 0.157) [51]. From a physiological point of view, the variations between individuals in lean mass gain are poorly understood, and the participation of different factors are mentioned: genetic, microRNA expression, the phosphorylation status of signaling proteins, androgen receptor concentrations, satellite cell count, mitochondrial function, and inflammatory cytokines [51,52]. In severe anorexic subjects (BMI: 12.6 ± 0.7 kg/m^2^), muscle atrophy has been described by MacLoughlin et al. according to a “metabolic myopathy” corresponding to type-2 fiber atrophy with abnormal accumulation of glycogen granules within fibers, which rapidly recovers as the nutrition improves [53,54].

On the other hand, muscle strength and endurance increased significantly in the trunk and limbs. Our results are difficult to compare with those of other studies due to different activities performed in populations of anorexic subjects of different ages and BMIs [47]. Only two medical teams were interested in developing strength during a physical activity program in anorexic patients [11,43,44]. Chantler et al. are the only ones who have studied a population comparable to ours in terms of average age (20 ± 5 years old), while Fernadez del Valle et al. studied 13–14 years old children [11,43,44]. Chantler et al. proposed muscle strengthening exercises against low resistance (2.5 kg dumbbells for the upper body, therapeutic elastic bands for the lower body, and body weight for the back) for 60 min twice a week for 8 weeks [44]. The absolute isokinetic strength of the knee extensors and flexors and the strength of the elbow flexors increased in 7 hospitalized anorexic subjects who ate 2500 calories per day and who had an average BMI of 15.1 ± 1.1 kg/m^2^ [44]. From a physiological point of view, several neural factors may be implied, including an increase in motor neuron excitability with inhibition of protective mechanisms to explain the increase in force [44]. This increase in strength was explained by the weight gain (+6 kg) during the refeeding protocol compared with control anorexic subjects (+3.3 kg). Re-nutrition responsible for weight gain was the main factor to explain the increase in strength, while the role of physical activities was difficult to isolate. More recently, Garrido et al. [12] showed that strength was significantly associated to BMI (r = 0.420; *p* < 0.01) before enteral refeeding in 23 severe restrictive anorexics aged 25.6 ± 6.2 years old (BMI: 11.4 ± 1.3 kg/m^2^). In the absence of physical activity, muscle strength, which had been manually assessed and summarily rated from 0 to 5, increased in 5 weeks in the trunk and the limbs as well as the body-weight [12]. Unfortunately, no association was sought based on the observed gains. When weight and strength gains were taken into account, we showed that the association was weak between isokinetic strength gain measured at the knee and weight gain. In addition, no significant association was found between isometric strength gain measured at the trunk and the lower limbs or the hand grip. This result suggested a specific role of the practice of adapted physical activities to increase strength. However, we could not confirm this due to the absence of control group.

Concerning bone parameters, it was not surprising to find that the bone mineral content remained unchanged in such a short period of physical activity [55,56]. Moreover, in anorexic subjects, the practice of high-impact physical activities such as repeated jumps was not enough to modify the markers of bone turnover (Bone-specific alkaline phosphatase, N-telopeptide, Osteocalcin) during intensive refeeding [57].

Another goal of physical activities was to improve function. The walking perimeter improved according to our results. We did not find any study that had analyzed this parameter in anorexic subjects, while the expected normal value in healthy subjects was 804 m according to the formula of Enright et al., which includes weight, height, and age [58]. Our population presented impaired walking at entry into PMR, which improved, on average, by 90 m but did not reach the expected standard. Fernandez del Valle et al. [43] studied the interest in 2010 of outpatient resistance training in children aged 14 years old on functional capacity according to the Time Up and Go test of 3 and 10 m and the Time Up and Down Stairs test. Despite an increase in strength, no functional gain was reported, probably due to already normal functional tests before training.

Balance also improved, probably due to a deficit at the entry into hospitalization (186 ± 86 mm with eyes open and 424 ± 165 mm with eyes closed). Indeed, balance with open and closed eyes was altered compared with the results of Fontana et al. [59], who evaluated a population of 15 anorexic subjects comparable to ours (age: 32 ± 11 years and BMI: 15.8 ± 1.8 kg/m^2^), hospitalized for a rehabilitation program, including physiotherapy, nutritional and psychological counseling. After our physical activity program, the values were comparable (122 ±161 mm) to those obtained by Fontana et al. eyes open (139 ± 87 mm; healthy subject: 104 ± 30 mm). On the other hand, the values obtained with eyes closed remained high (272 ± 164 mm vs. 160 ± 73 mm; healthy subjects: 124 ± 73 mm) [59]. This altered balance eyes closed may have been related to a state of anxiety present during the test, while strength gains in the lower limbs and trunk may have explained the balance gains. Indeed, anxiety and emotional factors can have a negative influence on the efficiency of human posture control due to interaction between vision and vestibular and somatosensory inputs [59]. In addition, a large variation between individuals was present, evidenced by the standard deviations of the values we reported.

Respiratory capacity also improved. Several studies have shown an alteration of respiratory capacities in anorexic subjects nicknamed “nutritional emphysema-like” [60,61,62]. However, ventilatory parameters usually remain within the norm, so there is no real “emphysema” [60]. In fact, these parameters undoubtedly depend on the degree of undernutrition. The lower the BMI of the anorexic population is, the lower the respiratory values are. For an average BMI of 18 kg/m^2^, the FEV1 and FVC are 106 and 109% of the predicted values, respectively [61], 106 and 100% if the average BMI is 16 kg/m^2^ [62], and 91 and 85% if the average BMI is 14.3 kg/m^2^ [60]. Before the stay in PMR, we reported values of 82% for a BMI of 13.8 kg/m^2^ with an improvement to 89 and 90% when the BMI improved to 14.8 kg/m^2^. Therefore, refeeding improved ventilatory parameters. Minano Garrido et al. [12] also showed a significant link between BMI and FEV1 (r = 0.660; *p* < 0.01). However, the practice of physical activities undoubtedly also contributed to ventilatory gain due to work based on the control of breath, with possibly an improvement in anxiety, independently of the weight gain. The improvement in muscle strength contributed to the improvement in respiratory capacities, as already demonstrated by Murciano et al. at the level of the diaphragm during refeeding [63]. The strength improvement of the intercostal muscles and, especially the abdominal muscles, due to an isometric strength gain of 48% during the abdominal Shirado test, undoubtedly contributed to a decrease in the residual volume, evaluated by Pieters et al., to 162% of the predicted values, explaining the use of the term “emphysema-like” [60].

The comparison between hyperactive and non-hyperactive anorexic subjects reported no difference for any of the measured parameters. Therefore, it seems possible to offer adapted and supervised physical activities during inpatient refeeding by nasogastric tube to anorexic subjects with hyperactivity. Few studies have attempted this experience because, most often, the subjects described in the literature are adolescents and present restrictive anorexia [11,42,43,45] or other eating disorders such as bulimia nervosa or Eating Disorder Not Otherwise Specified (EDNOS) [64]. Calogero et al. were, to our knowledge, the first to offer controlled physical activities to treat hyperactivity in patients with eating disorders [21], including multiple supervised exercises including stretching, yoga, Pilates, partner exercises, strength training, aerobic activities, and recreational games, were performed 1 h per session 4 times a week for 6 months. Weight improved by 43% and 38% in restrictive anorexic and bulimic patients, respectively. From a psychological point of view, monitored activities decreased anxiety and increased comfort with gaining weight, reduced anxiety during mealtimes, and increased compliance with the nutritive program [21]. Monitoring and individualizing the practice of physical activities according to weight gain has undoubtedly been successful. Thus, the weight gains were identical between hyperactive and non-hyperactive patients.

However, our results are questionable due to the premature end of the program for five patients, the dissatisfaction of some patients, and the relapses (21%). The three deaths are, of course, not attributable to the program but to the severity of the disease. More positively, 46% of patients continued to practice moderate physical activity, which testifies to the impact of educational advice. Unfortunately, two patients remained hyperactive, highlighting the difficulties in managing anorexia.

Our study has limits, especially concerning the patients’ selection, because only the most motivated by this type of PMR care were selected after psychiatric evaluation [21]. In addition, the absence of a control group does not allow us to assess exactly the role of physical activities in weight gain and other parameters improvement [23]. An RCT-type study would have been ideal but would present difficulties related to the definition of hyperactivity in anorexic patients and to the multitude of physical activities that could be offered according to durations, intensities, and frequencies. Indeed, the content of a physical activity program offered to anorexic children should probably be different from the program offered to adolescents or adults [42]. However, our adapted program followed some guidelines described by Cook et al. concerning progression, supervision, individualization, and monitoring according to energy expenditure and calorie intake [65]. The objectives were to gain strength and autonomy while allowing refeeding by nasogastric tube and psychiatric care. The evaluated parameters could have been different, for example, the body image or the evolution of patient anxiety [66].

## 5. Conclusions

The early return to controlled physical activities in PMR hospitalization did not compromise the efficiency of intensive refeeding in severe anorexia nervosa patients. It allowed an increase in functional performance with a rather good rate of patient satisfaction. However, a very attentive multidisciplinary approach was required in order not to compromise the efficiency of intensive refeeding by nasogastric tube. Further attention should be paid to the program to increase the long-term activity continuation and to avoid the premature ending of the hospitalization or disease relapse.

## Figures and Tables

**Table 1:** Initial bone mineral density (BMD).

Localisation	BMD	Mean (SD)	Min	Max
L1-L4 Spine	T-score	−2.76 ± 1.53	−5.4	0.4
	Z-score	−1.81 ± 1.29	−4.6	0.8
Femoral Neck	T-score	−2.21 ± 1.16	−4.1	0.2
	Z-score	−1.54 ± 1.10	−3.6	0.7
Trochanter	T-score	−2.48 ± 1.18	−4.7	−0.4
	Z-score	−1.80 ± 1.14	−4.1	0.2
Total Body	T-score	−1.51 ± 1.40	−4.2	0.7
	Z-score	−0.57 ± 1.22	−3.3	1.4

Abbreviations: BMD: Bone mineral density; Min: minimum value; Max: maximal value; SD: standard deviation; L: lumbar spine. Z-score: the number of standard deviations above or below the mean for the patient’s age, sex, and ethnicity. T-score: the number of standard deviations above or below the mean for a healthy 30-year-old adult of the same sex and ethnicity as the patient. Normal is a T-score of −1.0 or higher, Osteopenia is defined as between −1.0 and −2.5, and Osteoporosis is defined as −2.5 or lower.

**Table 2:** Biological parameters at the beginning of the PMR hospitalization.

Biological Parameters	Mean	Min	Max
Total Calcium (mmol/L)	2.30 ± 0.09	2.07	2.54
Phosphor (mmol/L)	1.20 ± 0.22	0.80	1.69
Proteins (mmol/L)	63.6 ± 5.7	48	74
D vitamin (N: 30–60 mg/L)	39.2 ± 24.9	6.4	142
Parathormone (N: 30–60 ui/L)	27.9 ± 8.6	15	47.9
Pal (N: 0–120 ui/L)	69.9 ± 52.1	24	250
ALAT (N < 36 ui/L)	45.9 ± 31.7	10	158
ASAT (N < 36 ui/L)	29.8 ± 17.9	12	94

Abbreviations: ALAT: Alanine Transferases; ASAT: Aspartate Amino Transferase; Pal: alkaline phosphatase; N: normal value.

**Table 3:** Fat mass, lean mass, and mineral content evolution (Body Dual X-ray Absorptiometry).

	Mass	PMR Entry	PMR Discharge	*p*	Gain (%)	95% CI
Total Body	Fat mass (kg)	2.49 ± 1.78	4.22 ± 3.52	0.02	1.73 ± 2.57 (69%)	0.9–2.6
	Lean mass (kg)	32.6 ± 3.0	32.9 ± 3.8	0.70	0.2 ± 2.2 (1%)	−0.5–0.9
	BMC (kg)	1.71 ± 0.37	1.61 ± 0.38	0.21	−0.09 ± 0.28 (−5%)	−0.6–0.8
Upper limbs	Fat mass (g)	190.0 ± 162.0	245.0 ± 186.0	0.01	55.0 ± 52.0 (29%)	37.7–72.3
	Lean mass (g)	3139.0 ± 534.0	3295.0 ± 602.0	0.23	160.0 ± 370.0 (5%)	36.6–283.4
	BMC (g)	231.0 ± 53.0	232.0 ± 48.0	0.89	0.78 ± 16.0 (0%)	−4.6–6.1
Lower limbs	Fat mass (g)	1009.0 ± 981.0	1490.0 ± 1078.0	0.002	481.0 ± 332.0 (47%)	370.3–591.7
	Lean mass (g)	10724.0 ± 1579.0	11045.0 ± 1776.0	0.29	320.0 ± 864.0 (2.9%)	31.9–608.0
	BMC (g)	671.0 ± 204.0	658.0 ± 188.0	0.44	−12.7 ± 47.0 (−2%)	−3.0–28.4
Trunk	Fat mass (g)	1150.1 ± 1041.0	1621.0 ± 1313.0	0.04	470.0 ± 579.0 (40%)	279.9–663.0
	Lean mass (g)	16256.0 ± 2261.0	16197.0 ± 1945.0	0.91	−59.0 ± 1512.0 (−1%)	−445.1–563.1
	BMC (g)	396.0 ± 124.0	387.0 ± 129.0	0.61	−9.2 ± 52.0 (−1%)	−8.1–26.5

Abbreviations: BMC: Bone Mineral content; PMR: Physical Medicine and Rehabilitation.

**Table 4:** Limb and trunk strength, balance, walking, and respiratory evolution during PMR care.

	PMR Entry	PMR Discharge	*p*	Gain (%)
Biceps isometric (kg)	0.31 ± 0.12	0.42 ± 0.16	0.0001	24.8
Triceps isometric (kg)	0.21 ± 0.11	0.27 ± 0.11	0.0001	21.2
Grip (kg)	0.63 ± 0.13	0.67 ± 0.12	0.006	4.6
Quadriceps isometric (kg)	0.41 ± 0.20	0.58 ± 0.22	0.0001	27.8
Quadriceps isokinetic (kg)	1.37 ± 0.34	1.53 ± 0.33	0.001	8.2
Hamstring isometric (kg)	0.29 ± 0.15	0.40 ± 0.16	0.0001	26.7
Hamstring isokinetic (kg)	0.96 ± 0.23	1.03 ± 0.21	0.005	7.4
Abdominal Shirado–Ito test (s)	56.0 ± 61.0	111.0 ± 56.0	0.0001	47.9
Spine Biering–Sorensen test (s)	95.0 ± 67.0	128.0 ± 54.0	0.001	25.9
Balance open eyes (mm)	186.0 ± 86.0	122.0 ± 161.0	0.07	34.0
Balance closed eyes (mm)	424.0 ± 165.0	272.0 ± 164.0	0.001	55.8
6-min walking test (m)	526.0 ± 143.0	617.0 ± 121.0	0.007	17.0
FVC (L) % predicted value	2.8 ± 0.57 82.0 ± 17.0	3.02 ± 0.64 89.0 ± 16.0	0.01 0.02	8.0 7.0
FEV1 (L/s) % predicted value	2.46 ± 0.60 82.0 ± 16.0	2.62 ± 0.61 90.0 ± 17.0	0.02 0.02	6.0 8.0
FEV1/FVC (%)	87.4 ± 1.25	87.0 ± 0.09	0.79	0.0

Abbreviations: PMR: Physical Medicine and Rehabilitation; FVC: Forced Vital Capacity; FEV1: Forced Expiratory Volume in one second.

**Table 5:** Comparison between hyperactive and non-hyperactive patients concerning body weight and BMI.

	Intensive Refeeding Care Entry	PMR Entry	PMR Discharge
Body weight (kg)			
- Hyperactive (*n* = 17)	33.6 ± 5.7	36.4 ± 7.0	38.7 ± 6.1
- Non hyperactive (*n* = 20)	34.3 ± 5.1	37.2 ± 3.6	40.1 ± 3.3
Body Mass Index (kg/m^2^)			
- Hyperactive (*n* = 17)	12.8 ± 1.2	13.8 ± 1.7	14.7 ± 1.4
- Non hyperactive (*n* = 20)	12.5 ± 1.8	13.6 ± 1.0	14.6 ± 0.9

Abbreviations: PMR: Physical Medicine and Rehabilitation.

## Data Availability

The data presented in this study are available on request from the corresponding author. The data are not publicly available due to ethical reasons.
